# Editorial: Women in science: neuropathic pain

**DOI:** 10.3389/fpain.2023.1273636

**Published:** 2023-08-22

**Authors:** Isabel Martins, Judith P. Golden, Fani Lourença Neto

**Affiliations:** ^1^ Instituto de Investigação e Inovação em Saúde da Universidade do Porto (I3S), Porto, Portugal; ^2^ Instituto de Biologia Molecular e Celular (IBMC), Universidade do Porto, Porto, Portugal; ^3^ Departamento de Biomedicina—Unidade de Biologia Experimental, Faculdade de Medicina, Universidade do Porto, Porto, Portugal; ^4^ Washington University Pain Center and Department of Anesthesiology, Washington University School of Medicine, St. Louis, MO, United States

**Keywords:** neuropathic pain, endocannabinoids, small fiber neuropathies, neurogenic rosacea, vulnerable populations

**Editorial on the Research Topic** Women in science: neuropathic pain

Chronic pain is a significant public health burden worldwide. According to the Center for Disease Control and Prevention, the prevalence of chronic pain, defined as pain lasting greater than three months is 20.4% of the adult population in the US (1). Those suffering from neuropathic pain, defined as pain resulting from damage to the peripheral or central nervous system, comprise a significant percentage of those with chronic pain (2). A number of diverse etiologies result in damage to the nervous system and the expression of neuropathic pain. These include diabetes, trauma, stroke, infections and chemotherapeutic agents (3–5). Several studies using telephone, computer or mail surveys or clinical examination have estimated the prevalence of neuropathic pain at 10% of the US adult population (6, 7). A number of studies have found a similar prevalence of neuropathic pain in other parts of the world (8–11), although there is considerable variability among some reports (2). The prevalence of neuropathic pain is not uniform across demographic groups and is reportedly higher in a number of subpopulations including women and those with lower household incomes (8, 9, 11). In the US, the cost attributed to the management of chronic pain conditions is estimated to be over $200 billion a year in combined direct health care expenditures and loss of productivity (12). Beyond the economic burden, the negative impact of neuropathic pain on the quality of life of patients and the suffering of those affected cannot be easily quantified (12–14). Neuropathic pain is reportedly of higher intensity and duration than non-neuropathic pain and is difficult to treat (5, 9, 15). In addition, many patients with neuropathic pain also experience other debilitating symptoms, which may include anxiety, depression, and sleep disturbances (3, 5, 13, 14). Current pharmacological treatments for neuropathic pain are sub-optimal and significant adverse effects limit the use of many therapies (5, 9, 13). The need to develop better treatments for neuropathic pain and comorbidities and to more efficiently target resources to disproportionately affected populations is therefore great.

This research topic, “Women in Science: Neuropathic Pain”, highlights recent work in which women scientists who are dedicated to studying neuropathic pain and other painful conditions have had a crucial role in the research team. The collection includes a set of manuscripts addressing several important issues related to neuropathic pain including the identification of demographic groups at greater risk for developing neuropathic pain, identifying disease conditions with a neuropathic pain component, and identifying mechanisms which underlie neuropathic pain conditions. In their original research article, Liktor-Busa et al. add new data to the growing body of information linking a deficiency in the endocannabinoid system to neuropathic pain. The authors found reduced levels of the endocannabinoid 2-arachidonoylglycerol associated with its increased hydrolysis in the periaqueductal gray, a critical area involved in descending pain modulation, in a rodent model of headache. Furthermore, they show that the inhibition of the 2-arachidonoylglycerol hydrolyzing enzymes, α/β-hydrolase domain-containing 6 and monoacylglycerol lipase, constitutes a potential therapeutic intervention for the treatment of headache. An area of research that has gained significant attention more recently is sensitive skin syndrome. The multifactorial origin of sensitive skin syndrome has hindered the establishment of its definitive underlying pathophysiology. Growing evidence indicates that one of the underlying causes of this condition involves a neurosensory dysfunction with a neuropathic component. Misery et al. review the recent literature concerning this type of small fiber neuropathy, by addressing the clinical and epidemiological data, assessment and diagnosis, pathophysiology, the role of environmental factors and the management of this condition. In line with the difficulty of classifying sensitive skin disorders as small fiber neuropathies, Li et al. draw attention to neurogenic rosacea, which is characterized by recurrent flushing and erythema in the central facial area. This condition also presents with many clinical manifestations similar to small fiber neuropathies. In their opinion article, Li et al. highlight these parallel manifestations and suggest that neurogenic rosacea could be a small fiber neuropathy. In another review article included in the collection, Rintoul et al. address how psychiatric conditions in the homeless and marginally housed population impact their pain experience. Several factors may contribute to aggravate pain, including psychiatric disorders such as anxiety, depression and post-traumatic stress. Both depression and anxiety are well-known pain comorbidities in clinical settings and their effects on chronic pain have been well established in preclinical models. Less understood is pain and the inherent comorbid conditions within specific vulnerable populations.

In conclusion, the manuscripts presented in this topic address a range of important issues regarding neuropathic pain which merit further investigation. It is our hope that further attention to these and other issues related to neuropathic pain will improve outcomes for all patients suffering from these debilitating conditions.
